# Supplementary material for: Phenolic compounds of Theobroma cacao L. show potential against dengue RdRp protease enzyme inhibition by In-silico docking, DFT study, MD simulation and MMGBSA calculation
Source: PLoS One. 2024 Mar 14;19(3):e0299238. doi: 10.1371/journal.pone.0299238 (PMC10939188; doi:10.1371/journal.pone.0299238)
Supplement: S1 Table — (DOCX) [file pone.0299238.s001.docx]

**S1 Table** Results of the phenolic compounds of *Theobroma cacao* L. against DENV-3 NS5 RdRp protein with their respective docking energy value and interacting residue in the binding site.

| **Sl. No.** | **Compound Name** | | **Interaction Energy (-kcal/mol)** | | **Amino acid residues** |
| --- | --- | --- | --- | --- | --- |
| 1 | Panduratin A | | -37.4166 | | Asp808, Trp833 (H-B), Asp808 (pi-anion), Trp833, Met809, Tyr882, Cys780 (alkyl/pi-alkyl), |
| 2 | (+)-Catechin | | -42.3027 | | Gln760, Asn777, Cys780, Val785 (H-B), Thr806 (C-H), Cys780 (pi-sulfur), Tyr882 (pi-pi stacked), Met809, Tyr882, Val785 (pi-alkyl) |
| 3 | Amentoflavone | | -50.8888 | | Lys756, Thr806 (H-B), Trp833, Met809 (C-H), Asp808 (pi-anion), Cys780 (pi-sulfur), Glu807, Tyr882 (pi-pi stacked/ amide pi-stacked), Val785, Met809 (pi-alkyl) |
| 4 | Apigenin 7-O-glucoside | | -55.0647 | | Ser776, Asn777, Ala759, Cys780, Asp808 (H-B), Lys756, Asn777, Asp808 (C-H), Asp808 (pi-anion), Cys780, Met809 (pi-alkyl) |
| 5 | Apigenin | | -36.1565 | | Lys756, Asn777, Val785 (H-B), Ala763, Cys780, Val785, Met809 (pi-alkyl) |
| 6 | Caffeic acid | | -28.9931 | | Lys756, Asn777, Thr806 (H-B), Cys780 (pi-sulfur), Tyr882 (pi-pi stacked), Met809 (pi-alkyl) |
| 7 | Chlorogenic acid | | -46.6545 | | Lys756, Val785, Thr806, Asp808, Trp833, Thr882 (H-B), Trp833 (C-H), Cys780 (pi-sulfur), Met809, Trp833 (pi-alkyl) |
| 8 | Coumaric acid | | -27.6924 | | Lys756, Asn777, Glu807 (H-B), Thr806 (C-H), 9Cys780 (pi-sulfur), Tyr882 (pi-pi stacked), Met809 (pi-alkyl) |
| 9 | Ferulic acid | | -28.2993 | | Lys756, Asp808, Trp833 (H-B), Thr806 (C-H), Met809 (pi-alkyl) |
| 10 | Gallic acid | | -26.714 | | Ser776, Asn777, Asp808, Trp833 (H-B), Asn777 (C-H), Cys780 (pi-sulfur), Met809 (pi-alkyl) |
| 11 | Hyperoside | | -47.0505 | | Lys756, Asn777, Cys780 (H-B), Asp808 (C-H), Ser776, Cys780 (pi-sulfur), Tyr882 (pi-pi staced/pi-pi T-shaped), Val785, Met809 (pi-alkyl) |
| 12 | Isoorientin | | -44.6763 | | Lys756, Asn777, Cys780, Val785, Thr806 (H-B), Cys780 (pi-sulfur), Tyr882 (pi-pi stacked), Met809 (pi-alyl) |
| 13 | Isorhamnetin | | -39.9978 | | Lys756, Asn777, Val785, Asp808, Trp833 (H-B), Thr806, Met809 (C-H), Ala763, Cys780, Val785, Met809 (alkyl/pi-alkyl) |
| 14 | Isorhoifolin | | -57.259 | | Lys756, Gln760, Asp808, Thr805, Tyr882 (H-B), Thr806, Glu807 (C-H), Tyr882 (pi-sigma), Trp833 (pi-pi stacked), Ala763, Val785, Cys780, Met809 (alkyl/pi-alkyl) |
| 15 | Isovitexin | | -49.6822 | | Ser776, Cys780, Gln760, Thr805, Lys756, Tyr882, Asp808, Trp833 (H-B), Met809, Thr806, Lys756, Gln760 (C-H), Trp833 (pi-pi stacked), Ala763, Cys780 (pi-alkyl) |
| 16 | Kaempferol 3-O-β-D-glucoside | | -49.5049 | | Ser776, Asn777, Gln760, Lys756, Thr805, Val785, Tyr882 (H-B), Glu807, Asp808, Thr809 (C-H), Cys780 (pi-sulfur), Asp808 (pi-anion), Tyr882 (pi-pi stacked), Ala763, Met809 (pi-alkyl) |
| 17 | Kaempferol-7-  Oneohesperidoside | | -51.8406 | | Gln760, Ser776, Val785, Thr806, Tyr882 (H-B), Asn777, Lys756, Tyr882, Thr806 (C-H) |
| 18 | Luteolin | | -39.287 | | Lys756, Asn777, Ser776 (H-B), Lys756 (C-H), Cys780 (pi-sulfur), Ala763, Met809 (pi-alkyl) |
| 19 | Naringenin | | -34.7134 | | Lys756, Asn777 (H-B), Thr806, Tyr882 (C-H), Cys780 (pi-sulfur), Tyr882 (pi-pi stacked), Val785, Met809 (pi-alkyl) |
| 20 | Naringin | | -60.2921 | | Lys756, Gln760, Ser776, Asn777, Val785, Thr806, Tyr882 (H-B), Asn777, Thr806, Tyr882 (C-H), Asp808 (pi-anion) |
| 21 | Nicotiflorin | | -54.5033 | | Ser776, Asn777, Lys756, Gln760, Met809, Tyr882 (H-B), Thr806, Val785, Tyr882 (C-H), Trp833 (pi-pi T-shaped), Ala763, Cys780, Val785, Leu8120 (alkyl/pi-alkyl) |
| 22 | Orientin | | -51.365 | | Ser776, Asn777, Gln760, Asp808, Thr806, Asp881, Trp833 (H-B), Met809, Asp881 (C-H), Cys780 (pi-sulfur), Trp833 (pi-pi stacked), Ala763 (pi-alkyl) |
| 23 | Protocatechuic acid | | -25.3796 | | Ser776, Asn777, Cys780, Asp808, Trp833 (H-B), Cys780 (pi-sulfur), Met809 (pi-alkyl) |
| 24 | Prunin | | -54.6302 | | Ser776, Asn777, Cys780, Ala759, Asp808, Val785 (H-B), Asn777 (C-H), Asp808 (pi-anion), Cys780 (pi-sulfur), Glu807 (amide-pi stacked), Met809 (pi-alkyl) |
| 25 | Quercetin | | -40.2612 | | Asn777, Cys780, Gln760, Lys756 (H-B), Cys780 (pi-sulfur), Tyr882 (pi-pi stacked), Val785, Met809 (pi-alkyl) |
| 26 | Quercitrin | -48.564 | | Ser776, Asn777, Cys780, Gln760, Thr805, Lys756, Val785, Tyr882 (H-B), Thr806, Glu807, Asp808 (C-H), Asp808 (pi-anion), Tyr882 (pi-pi T-shaped), Ala763, Met809, Cys780, Val785 (alkyl/pi-alkyl) | |
| 27 | Rutin | -60.4769 | | Ser776, Asn777, Cys780, Gln760, Thr805, Val785, Tyr882 (H-B), Lys756, Gln760, Thr806, Asn777, Tyr882 (C-H), Asp808 (pi-anion), Trp833, Thr882 (pi-alkyl) | |
| 28 | Luteolin 7-O-β-D-glucoside | -44.0467 | | Asn777, Cys780, Gln760, Val785, Glu807 (H-B), Thr806, Tyr882 (C-H), Cys780 (pi-sulfur), Tyr882 (pi-pi stacked), Val785, Met809 (pi-alkyl) | |
| 29 | Isoquercetin | -44.535 | | Lys756, Cys780, Ser776, Asn777, Val785, Thr806, Trp833 (H-B), Asn777 (C-H) | |
| 30 | Epicatechin | -24.264 | | Asn777, Cys780, Met809 (H-B), Asp808, Met809 (C-H), Cys780 (pi-sulfur), Tyr882 (pi-pi stacked), Cys780, Met809, Trp833, Tyr882 (pi-alkyl) | |
