# Supplementary material for: Phenolic compounds of Theobroma cacao L. show potential against dengue RdRp protease enzyme inhibition by In-silico docking, DFT study, MD simulation and MMGBSA calculation
Source: PLoS One. 2024 Mar 14;19(3):e0299238. doi: 10.1371/journal.pone.0299238 (PMC10939188; doi:10.1371/journal.pone.0299238)
Supplement: S2 Table — (DOCX) [file pone.0299238.s002.docx]

**S2 Table.** **Molecular physicochemical descriptors and drug-likeness analysis of the selected compounds.**

| **Compound Name** | **Physicochemical Properties** | | | | | | **Druglikeness** | | | | | | | | | | |
| --- | --- | --- | --- | --- | --- | --- | --- | --- | --- | --- | --- | --- | --- | --- | --- | --- | --- |
|  | **MW** | **M. R.** | **TPSA** | **H-A** | **H-D** | **N. R.** | **Lipinski** | | **Ghose** | | **Veber** | | **Egan** | | **Muegge** | | **B. S.** |
|  |  |  |  |  |  |  | Pr | Vi | Pr | Vi | Pr | Vi | Pr | Vi | Pr | Vi |  |
| Panduratin A | 406.51 | 121.48 | 66.76 | 4 | 2 | 6 | Yes | 0 | No | 1 | Yes | 0 | No | 1 | No | 1 | 0.55 |
| (+)-Catechin | 290.27 | 74.33 | 110.38 | 6 | 5 | 1 | Yes | 0 | Yes | 0 | Yes | 0 | Yes | 0 | Yes | 0 | 0.55 |
| Amentoflavone | 538.46 | 146.97 | 181.80 | 10 | 6 | 3 | No | 2 | No | 2 | No | 1 | No | 1 | No | 3 | 0.17 |
| Apigenin 7-O-glucoside | 432.38 | 106.11 | 170.05 | 10 | 6 | 4 | Yes | 1 | Yes | 0 | No | 1 | No | 1 | No | 2 | 0.55 |
| Chlorogenic acid | 354.31 | 83.50 | 164.75 | 9 | 6 | 5 | Yes | 1 | No | 1 | No | 1 | No | 1 | No | 2 | 0.11 |
| Hyperoside | 464.38 | 10.16 | 210.51 | 12 | 8 | 4 | No | 2 | No | 1 | No | 1 | No | 1 | No | 3 | 0.17 |
| Isoorientin | 448.38 | 108.63 | 201.28 | 11 | 8 | 3 | No | 2 | No | 1 | No | 1 | No | 1 | No | 3 | 0.17 |
| Isorhamnetin | 316.26 | 82.50 | 120.36 | 7 | 4 | 2 | Yes | 0 | Yes | 0 | Yes | 0 | Yes | 0 | Yes | 0 | 0.55 |
| Isorhoifolin | 578.52 | 137.33 | 228.97 | 14 | 8 | 6 | No | 3 | No | 4 | No | 1 | No | 1 | No | 3 | 0.17 |
| Isovitexin | 432.38 | 106.61 | 181.05 | 10 | 7 | 3 | Yes | 1 | Yes | 0 | No | 1 | No | 1 | No | 2 | 0.55 |
| Kaempferol 3-O-β-D-glucoside | 447.37 | 106.24 | 193.11 | 11 | 6 | 4 | No | 2 | Yes | 0 | No | 1 | No | 1 | No | 3 | 0.11 |
| Kaempferol-7-Oneohesperidoside | 594.52 | 139.36 | 249.20 | 15 | 9 | 6 | No | 3 | No | 4 | No | 1 | No | 1 | No | 3 | 0.17 |
| Luteolin | 286.24 | 76.01 | 111.13 | 6 | 4 | 1 | Yes | 0 | Yes | 0 | Yes | 0 | Yes | 0 | Yes | 0 | 0.55 |
| Naringin | 580.53 | 134.91 | 225.06 | 14 | 8 | 6 | No | 3 | No | 4 | No | 1 | No | 1 | No | 3 | 0.17 |
| Nicotiflorin | 594.52 | 139.36 | 249.20 | 15 | 9 | 6 | No | 3 | No | 4 | No | 1 | No | 1 | No | 3 | 0.17 |
| Orientin | 448.38 | 108.63 | 201.28 | 11 | 8 | 3 | No | 2 | No | 1 | No | 1 | No | 1 | No | 3 | 0.17 |
| Prunin | 434.39 | 103.69 | 166.14 | 10 | 6 | 4 | Yes | 1 | Yes | 0 | No | 1 | No | 1 | No | 2 | 0.55 |
| Quercetin | 302.24 | 78.03 | 131.36 | 7 | 5 | 1 | Yes | 0 | Yes | 0 | Yes | 0 | Yes | 0 | Yes | 0 | 0.55 |
| Quercitrin | 448.38 | 109.00 | 190.28 | 11 | 7 | 3 | Yes | 2 | Yes | 0 | No | 1 | No | 1 | No | 3 | 0.17 |
| Rutin | 610.52 | 141.38 | 269.43 | 16 | 10 | 6 | No | 3 | No | 4 | No | 1 | No | 1 | No | 4 | 0.17 |
| Luteolin 7-O-β-D-glucoside | 448.38 | 108.13 | 190.28 | 11 | 7 | 4 | No | 2 | Yes | 0 | No | 1 | No | 1 | No | 3 | 0.17 |
| Isoquercetin | 464.38 | 110.16 | 210.51 | 12 | 8 | 4 | No | 2 | No | 1 | No | 1 | No | 1 | No | 3 | 0.17 |
