# Supplementary material for: Phenolic compounds of Theobroma cacao L. show potential against dengue RdRp protease enzyme inhibition by In-silico docking, DFT study, MD simulation and MMGBSA calculation
Source: PLoS One. 2024 Mar 14;19(3):e0299238. doi: 10.1371/journal.pone.0299238 (PMC10939188; doi:10.1371/journal.pone.0299238)
Supplement: S3 Table — (DOCX) [file pone.0299238.s003.docx]

**S3 Table.** **ADMET profiling enlisting absorption, distribution, metabolism and toxicity related drug-likeness parameters.**

| **Compound Name** | **WS** | **HIA** | **BBB** | **Substrate** | | **Inhibitor** | | | | | **AMES T.** | **hERG I** | **hERG II** | **HT** | **SS** |
| --- | --- | --- | --- | --- | --- | --- | --- | --- | --- | --- | --- | --- | --- | --- | --- |
|  |  |  |  | **CYP2D6** | **CYP3A4** | **CYP1A2** | **CYP2C19** | **CYP2C9** | **CYP2D6** | **CYP3A4** |  |  |  |  |  |
| Panduratin A | -5.262 | 95.369 | -0.476 | No | Yes | No | Yes | Yes | No | Yes | No | No | Yes | No | No |
| (+)-Catechin | -3.117 | 68.829 | -1.054 | No | No | No | No | No | No | No | No | No | No | No | No |
| Isorhamnetin | -3 | 76.014 | -1.135 | No | No | Yes | No | No | No | No | No | No | No | No | No |
| Luteolin | -3.094 | 81.13 | -0.907 | No | No | Yes | No | Yes | No | No | No | No | No | No | No |
| Quercetin | -2.925 | 77.207 | -1.098 | No | No | Yes | No | No | No | No | No | No | No | No | No |
