# Supplementary material for: Phenolic compounds of Theobroma cacao L. show potential against dengue RdRp protease enzyme inhibition by In-silico docking, DFT study, MD simulation and MMGBSA calculation
Source: PLoS One. 2024 Mar 14;19(3):e0299238. doi: 10.1371/journal.pone.0299238 (PMC10939188; doi:10.1371/journal.pone.0299238)
Supplement: S4 Table — (DOCX) [file pone.0299238.s004.docx]

**S4 Table.** **Showing properties of atoms of the compound and control.**

| **Element** | **hybridization** | **occupancy** | **Oxidation state** | **ESP Charge** | **Milliken charge** | **Hirshfeld charge** |
| --- | --- | --- | --- | --- | --- | --- |
| **(+)Catechin** | | | | | | |
| Oxygen  Oxygen  Oxygen  Oxygen  Oxygen  Oxygen  Carbon  Carbon  Carbon  Carbon  Carbon  Carbon  Carbon  Carbon  Carbon  Carbon  Carbon  Carbon  Carbon  Carbon  Carbon  Hydrogen  Hydrogen  Hydrogen  Hydrogen  Hydrogen  Hydrogen  Hydrogen  Hydrogen  Hydrogen  Hydrogen  Hydrogen  Hydrogen  Hydrogen  Hydrogen | Sp2  Sp3  Sp3  Sp3  Sp3  Sp3  Sp3  Sp3  Sp3  Sp2  Sp2  Sp2  Sp2  Sp2  Sp2  Sp2  Sp2  Sp2  Sp2  Sp2  Sp2  None  None  None  None  None  None  None  None  None  None  None  None  None  None | 1  1  1  1  1  1  1  1  1  1  1  1  1  1  1  1  1  1  1  1  1  1  1  1  1  1  1  1  1  1  1  1  1  1  1 | 0  0  0  0  0  0  0  0  0  0  0  0  0  0  0  0  0  0  0  0  0  0  0  0  0  0  0  0  0  0  0  0  0  0  0 | -0.526  -0.884  -0.773  -0.794  -0.783  -0.759  0.366  0.475  -0.225  -0.412  0.493  -0.245  0.708  -0.69  -0.319  -0.079  -0.775  0.772  0.438  -0.351  0.391  0.066  0.045  0.119  0.114  0.242  0.171  0.521  0.14  0.271  0.197  0.519  0.517  0.531  0.518 | -0.576  -0.644  -0.658  -0.655  -0.658  -0.653  0.11  0.038  -0.239  -0.024  0.333  0.077  0.32  -0.217  -0.165  -0.123  -0.21  0.329  0.291  -0.139  0.274  0.138  0.142  0.14  0.14  0.125  0.13  0.389  0.114  0.124  0.129  0.407  0.402  0.404  0.404 | -0.194  -0.29  -0.228  -0.239  -0.246  -0.246  0.082  0.086  -0.036  -0.036  0.071  -0.004  0.085  -0.087  -0.054  -0.042  -0.085  0.084  0.072  -0.049  0.072  0.028  0.032  0.025  0.03  0.044  0.047  0.177  0.045  0.046  0.054  0.193  0.191  0.192  0.192 |
| **Panduratin A** | | | | | | |
| Oxygen  Oxygen  Oxygen  Oxygen  Carbon  Carbon  Carbon  Carbon  Carbon  Carbon  Carbon  Carbon  Carbon  Carbon  Carbon  Carbon  Carbon  Carbon  Carbon  Carbon  Carbon  Carbon  Carbon  Carbon  Carbon  Carbon  Carbon  Carbon  Carbon  Carbon  Hydrogen  Hydrogen  Hydrogen  Hydrogen  Hydrogen  Hydrogen  Hydrogen  Hydrogen  Hydrogen  Hydrogen  Hydrogen  Hydrogen  Hydrogen  Hydrogen  Hydrogen  Hydrogen  Hydrogen  Hydrogen  Hydrogen  Hydrogen  Hydrogen  Hydrogen  Hydrogen  Hydrogen  Hydrogen  Hydrogen  Hydrogen  Hydrogen  Hydrogen  Hydrogen | Sp2  Sp3  Sp3  Sp3  Sp3  Sp3  Sp3  Sp3  Sp2  Sp3  Sp2  Sp2  Sp2  Sp3  Sp2  Sp2  Sp2  Sp2  Sp2  Sp2  Sp2  Sp2  Sp2  Sp2  Sp3  Sp3  Sp2  Sp2  Sp2  Sp3  None  None  None  None  None  None  None  None  None  None  None  None  None  None  None  None  None  None  None  None  None  None  None  None  None  None  None  None  None  None | 1  1  1  1  1  1  1  1  1  1  1  1  1  1  1  1  1  1  1  1  1  1  1  1  1  1  1  1  1  1  1  1  1  1  1  1  1  1  1  1  1  1  1  1  1  1  1  1  1  1  1  1  1  1  1  1  1  1  1  1 | 0  0  0  0  0  0  0  0  0  0  0  0  0  0  0  0  0  0  0  0  0  0  0  0  0  0  0  0  0  0  0  0  0  0  0  0  0  0  0  0  0  0  0  0  0  0  0  0  0  0  0  0  0  0  0  0  0  0  0  0 | -0.778  -0.825  -0.732  -0.553  -0.283  0.079  -0.307  -0.096  0.007  0.009  -0.229  0.263  1.27  -0.425  -0.353  -1.069  -0.208  -0.195  0.253  0.975  0.716  -0.134  -0.146  -0.157  -0.495  -0.449  -0.82  -0.586  0.808  0.027  0.116  0.067  0.112  0.099  0.078  0.013  0.06  0.132  0.122  0.134  0.127  0.127  0.139  0.136  0.135  0.138  0.134  0.26  0.171  0.145  0.132  0.14  0.121  0.125  0.142  0.518  0.523  0.096  0.11  0.082 | -0.537  -0.661  -0.644  -0.601  -0.159  -0.145  -0.142  -0.188  0.083  -0.221  -0.111  0.056  0.294  -0.32  -0.129  0.036  -0.107  -0.121  0.056  0.32  0.321  -0.112  -0.109  -0.106  -0.302  -0.339  -0.191  -0.228  0.374  -0.057  0.175  0.124  0.117  0.121  0.099  0.114  0.123  0.078  0.112  0.099  0.107  0.089  0.112  0.095  0.1  0.101  0.097  0.125  0.141  0.104  0.096  0.102  0.111  0.104  0.111  0.405  0.411  0.139  0.138  0.137 | -0.293  -0.239  -0.154  -0.183  -0.01  0.013  0.017  -0.033  0.005  -0.031  -0.05  0.014  0.177  -0.066  -0.055  -0.049  -0.041  -0.046  0.018  0.102  0.105  -0.039  -0.039  -0.044  -0.066  -0.068  -0.068  -0.084  0.099  0.036  0.003  0.014  0.012  0.012  0.019  0.013  0.023  0.023  0.025  0.027  0.027  0.018  0.03  0.022  0.037  0.038  0.036  0.045  0.045  0.025  0.026  0.019  0.017  0.023  0.028  0.137  0.199  0.047  0.054  0.046 |
