# Supplementary material for: Phenolic compounds of Theobroma cacao L. show potential against dengue RdRp protease enzyme inhibition by In-silico docking, DFT study, MD simulation and MMGBSA calculation
Source: PLoS One. 2024 Mar 14;19(3):e0299238. doi: 10.1371/journal.pone.0299238 (PMC10939188; doi:10.1371/journal.pone.0299238)
Supplement: S5 Table — (DOCX) [file pone.0299238.s005.docx]

**S5 Table.** **Showing properties of bonds of the compound and control.**

| **Name** | | **Bond Order** | **Bond Type** | **Bond length** | |
| --- | --- | --- | --- | --- | --- |
| **(+)Catechin** | | | | | |
| O1 - C2  O1 - C5  O2 - C1  O2 - H7  O3 - C7  O3 - H11  O4 - C12  O4 - H12  O5 - C13  O5 - H13  O6 - C15  O6 - H14  C1 - C2  C1 - C3  C1 - H1  C2 - C6  C2 - H2  C3 - C4  C3 - H3  C3 - H4  C4 - C5  C4 - C7  C5 - C8  C6 - C9  C6 - C10  C7 - C11  C8 - C12  C8 - H5  C9 - C13  C9 - H6  C10 - C14  C10 - H8  C11 - C12  C11 - H9  C13 - C15  C14 - C15  C14 - H10 | | 1  1  1  1  1  1  1  1  1  1  1  1  1  1  1  1  1  1  1  1  1  2  2  2  1  1  1  1  1  1  2  1  2  1  2  1  1 | Single  Single  Single  Single  Single  Single  Single  Single  Single  Single  Single  Single  Single  Single  Single  Single  Single  Single  Single  Single  Single  Double  Double  Double  Single  Single  Single  Single  Single  Single  Double  Single  Double  Single  Double  Single  Single | 1.43669  1.36926  1.42381  0.972452  1.36413  0.972512  1.36101  0.972573  1.3621  0.972684  1.36219  0.972601  1.53519  1.52625  1.09695  1.51757  1.09943  1.49862  1.09776  1.09701  1.39903  1.40144  1.39758  1.39297  1.39304  1.39492  1.39404  1.08764  1.39491  1.08699  1.39479  1.08652  1.39285  1.08735  1.39501  1.39477  1.08679 | |
| **Panduratin A** | | | | | |
| O1 - C9  O2 - C16  O2 - H26  O3 - C17  O3 - H27  O4 - C25  O4 - C26  C1 - C2  C1 - C3  C1 - C9  C1 - H1  C2 - C5  C2 - C6  C2 - H2  C3 - C4  C3 - C8  C3 - H3  C4 - C7  C4 - H4  C4 - H5  C5 - C7  C5 - C10  C6 - C11  C6 - H6  C6 - H7  C7 - H8  C8 - C13  C8 - C14  C9 - C12  C10 - H9  C10 - H10  C10 - H11  C11 - C15  C11 - H12  C12 - C16  C12 - C17  C13 - C18  C13 - H13  C14 - C19  C14 - H14  C15 - C21  C15 - C22  C16 - C23  C17 - C24  C18 - C20  C18 - H15  C19 - C20  C19 - H16  C20 - H17  C21 - H20  C21 - H21  C21 - H22  C22 - H23  C22 - H24  C22 - H25  C23 - C25  C23 - H18  C24 - C25  C24 - H19  C26 - H28  C26 - H29  C26 - H30 | 2  1  1  1  1  1  1  1  1  1  1  1  1  1  1  1  1  1  1  1  2  1  1  1  1  1  2  1  1  1  1  1  2  1  2  1  1  1  2  1  1  1  1  2  2  1  1  1  1  1  1  1  1  1  1  2  1  1  1  1  1  1 | | Double  Single  Single  Single  Single  Single  Single  Single  Single  Single  Single  Single  Single  Single  Single  Single  Single  Single  Single  Single  Double  Single  Single  Single  Single  Single  Double  Single  Single  Single  Single  Single  Double  Single  Double  Single  Single  Single  Double  Single  Single  Single  Single  Double  Double  Single  Single  Single  Single  Single  Single  Single  Single  Single  Single  Double  Single  Single  Single  Single  Single  Single | | 1.22885  1.36303  0.96997  1.36306  0.972464  1.36112  1.42677  1.55136  1.54667  1.50765  1.08435  1.52221  1.53695  1.10146  1.54517  1.52638  1.1009  1.50068  1.09704  1.09728  1.34503  1.50379  1.49547  1.09432  1.09686  1.08723  1.39121  1.39126  1.4903  1.09442  1.09545  1.09485  1.34645  1.08989  1.37913  1.37921  1.39496  1.08283  1.39491  1.08317  1.50559  1.50243  1.39481  1.39488  1.39491  1.08621  1.39489  1.08611  1.08603  1.09496  1.09515  1.09501  1.09428  1.09231  1.09529  1.39497  1.08715  1.39485  1.08491  1.0953  1.09495  1.0954 |
