# Supplementary material for: Phenolic compounds of Theobroma cacao L. show potential against dengue RdRp protease enzyme inhibition by In-silico docking, DFT study, MD simulation and MMGBSA calculation
Source: PLoS One. 2024 Mar 14;19(3):e0299238. doi: 10.1371/journal.pone.0299238 (PMC10939188; doi:10.1371/journal.pone.0299238)
Supplement: Attachment — Submitted filename: Dear Authors_6-12.pdf [file pone.0299238.s006.pdf]

Dear Authors,

First:

I have reviewed the section of your manuscript concerning the Molecular Dynamic (MD) simulations and would like to discuss the interpretation of the RMSD (Root Mean Square Deviation) and RMSF (Root Mean Square Fluctuation) values in relation to the stability of the (+)-Catechin-protein complex.

In your manuscript, you mention that the (+)-Catechin-protein complex exhibited stability throughout the 100 ns MD simulation period. However, I observed that the RMSD value for the complex increased significantly, reaching up to 25.5 Å, and the average RMSD was reported as 18.0 Å【17†source】. Typically, a lower RMSD value during a simulation indicates a stable protein-ligand complex, while a higher RMSD value suggests less stability.

Furthermore, the RMSF values, which reflect the flexibility and mobility of amino acid residues during the simulation, also showed considerable variation, with the average RMSF observed for RdRp following binding of (+)-Catechin being 10.0 Å【18†source】. Higher RMSF values typically indicate greater flexibility and potentially less stability in specific regions of the protein.

Given these observations, it seems that there is a contradiction in stating that the complex remains stable while the RMSD and RMSF values suggest significant fluctuations and changes in the structure. It would be beneficial for the manuscript if you could clarify the following points:

1. Interpretation of RMSD Values: How do you reconcile the relatively high RMSD values with the conclusion that the complex is stable? Is there a specific criterion or threshold you are using to define stability in this context?

2. RMSF Insights: Could you provide more insights into the high RMSF values observed? Are these fluctuations concentrated in specific regions of the protein, and do they affect the overall stability of the complex?

3. Impact on Binding Efficacy: How do these fluctuations influence the binding efficacy and potential inhibitory action of (+)-Catechin on the RdRp protease?

Your clarification on these points would greatly enhance the understanding of the MD simulation results and their implications for the stability and efficacy of the (+)-Catechin-protein complex as a potential DENV inhibitor.

Second:

I have carefully reviewed your manuscript, particularly the Molecular Dynamic (MD) simulations section, focusing on the RMSF (Root Mean Square Fluctuation) values of the (+)-Catechin-protein complex. One key aspect that appears to be missing in your analysis is the comparison of these RMSF values with the RMSF of the apo protein (the unbound state of the protein).

In your study, you report the RMSF values for the (+)-Catechin-protein complex, highlighting the flexibility and mobility of amino acid residues during the simulation. These values provide essential insights into the stability and conformational dynamics of the protein-ligand complex【18†source】. However, for a comprehensive understanding of how the binding of (+)-Catechin affects the protein, it

would be beneficial to compare these results with the RMSF of the protein in its unbound state. Such a comparison can offer valuable information on the following:

1. Effect of Ligand Binding on Protein Dynamics**: Comparing the RMSF values of the apo protein with the complex can reveal if and how the binding of (+)-Catechin alters the dynamic behavior of the protein. This can be particularly informative for understanding any conformational changes or stabilization effects induced by ligand binding.

2.Regions of Increased or Decreased Flexibility**: By contrasting the RMSF profiles of the apo and bound states, you can identify specific regions in the protein that become more or less flexible upon ligand binding. This can provide insights into the mechanism of inhibition and the structural basis of the protein's functional modulation.

3. Benchmark for Stability Analysis**: Comparing the RMSF values of the apo and bound states can serve as a benchmark to assess the relative stability imparted by ligand binding. This comparison could strengthen your conclusions about the stability and efficacy of the (+)-Catechin-protein complex as a potential inhibitor.

I believe that incorporating this comparative analysis would greatly enhance the depth and significance of your MD simulation results, offering a more nuanced understanding of the interaction dynamics between (+)-Catechin and the RdRp protease.

Third:

Regarding the section "3.5. Density Function Theory (DFT) Calculations" in your manuscript, I would like to offer a brief comment for improvement. While the results in Table 1 provide valuable data on the electronic properties of (+)-Catechin, there appears to be a missed opportunity in not further interpreting these results to deduce the nature of this compound in terms of its reactivity and interaction mechanism.

Your analysis effectively identifies the HOMO and LUMO energy levels, and the energy gap, but stops short of extrapolating how these properties translate into the compound's behavior as an electron donor or acceptor. Such an interpretation is crucial for understanding the molecular basis of its potential inhibitory action on the RdRp protease.

This additional step of analysis could significantly strengthen your DFT section, providing deeper insights into the electronic characteristics and reactivity of (+)-Catechin, and how these properties contribute to its function as a potential inhibitor. It would be beneficial to readers and the field if this aspect could be explored and discussed.

fourth:

I have reviewed your manuscript and would like to recommend the inclusion of some recent and relevant references that could significantly enhance the depth and context of your work. These references provide insights and data that align closely with your study's focus and could offer additional perspectives or comparative data to strengthen your arguments.

1. [Molecules: DOI 10.3390/molecules27196320]

2. Reference on Molecular Docking and Simulation Techniques:

[Crystals: DOI 10.3390/cryst13071086](https://doi.org/10.3390/cryst13071086)

3. Reference on Density Functional Theory (DFT) Analysis:

[Crystals: DOI 10.3390/cryst13071020]

This article covers the latest developments in DFT calculations, potentially providing a broader context or newer techniques that could be applied to your DFT analysis.
